# Respiratory Polygraphy Patterns and Risk of Recurrent Cardiovascular Events in Patients With Acute Coronary Syndrome

**DOI:** 10.3389/fmed.2022.870906

**Published:** 2022-06-27

**Authors:** Andrea Zapater, Geoffroy Solelhac, Alicia Sánchez-de-la-Torre, Esther Gracia-Lavedan, Ivan David Benitez, Gerard Torres, Jordi De Batlle, José Haba-Rubio, Mathieu Berger, Jorge Abad, Joaquín Duran-Cantolla, Amaia Urrutia, Olga Mediano, María José Masdeu, Estrella Ordax-Carbajo, Juan Fernando Masa, Mónica De la Peña, Mercé Mayos, Ramon Coloma, Josep María Montserrat, Eusebi Chiner, Olga Mínguez, Lydia Pascual, Anunciación Cortijo, Dolores Martínez, Mireia Dalmases, Chi-Hang Lee, R. Doug McEvoy, Ferran Barbé, Raphael Heinzer, Manuel Sánchez-de-la-Torre, Spanish Sleep Network, Laura Abad

**Affiliations:** ^1^Precision Medicine in Chronic Diseases, Hospital Universitari Arnau de Vilanova-Santa Maria, IRB Lleida, Department of Nursing and Physiotherapy, Faculty of Nursing and Physiotherapy, University of Lleida, Lleida, Spain; ^2^Centro de Investigación Biomédica en Red de Enfermedades Respiratorias (CIBERES), Madrid, Spain; ^3^Center for Investigation and Research in Sleep, Lausanne University Hospital and University of Lausanne, Lausanne, Switzerland; ^4^Translation Research in Respiratory Medicine, Hospital Universitari Arnau de Vilanova-Santa Maria, IRBLleida, Lleida, Spain; ^5^Respiratory Department, Hospital Universitari Germans Trias i Pujol, Barcelona, Spain; ^6^Servicio de Investigación OSI, IIS Bioaraba, Hospital Universitario Araba, Vitoria-Gasteiz, Spain; ^7^Servicio Neumologia, Hospital Universitario Cruces, Bizkaia, Spain; ^8^Respiratory Department, Hospital Universitario de Guadalajara, Guadalajara, Spain; ^9^Respiratory and Sleep Department, Hospital Universitari Parc Taulí, Institut Investigació i Innovació Parc Taulí (I3PT), Universitat Autònoma de Barcelona, Barcelona, Spain; ^10^Respiratory Department, Hospital Universitario de Burgos, Burgos, Spain; ^11^Respiratory Department, Hospital San Pedro Alcántara, Cáceres, Spain; ^12^Clinic Analysis and Respiratory Services, Institut de Investigació Sanitaria de Palma, Hospital Universitari Son Espases, Palma de Mallorca, Spain; ^13^Sleep Unit, Department of Respiratory Medicine, Hospital de la Santa Creu i Sant Pau, Barcelona, Spain; ^14^Respiratory Department, Hospital General Universitario de Albacete, Albacete, Spain; ^15^Respiratory Department, Hospital Clinic, Barcelona, Spain; ^16^Respiratory Department, Hospital Universitari Sant Joan d’Alacant, Alicante, Spain; ^17^Cardiovascular Research Institute, National University of Singapore, Singapore, Singapore; ^18^Adelaide Institute for Sleep Health, College of Medicine and Public Health, Flinders University, Adelaide, SA, Australia; ^19^Pulmonary Department, Lausanne University Hospital and University of Lausanne, Lausanne, Switzerland

**Keywords:** acute coronary syndrome, cardiovascular disease, obstructive sleep apnea, precision medicine, respiratory polygraphy

## Abstract

**Introduction:**

Obstructive sleep apnea (OSA) severity is based on the apnea-hypopnea index (AHI). The AHI is a simplistic measure that is inadequate for capturing disease severity and its consequences in cardiovascular diseases (CVDs). Deleterious effects of OSA have been suggested to influence the prognosis of specific endotypes of patients with acute coronary syndrome (ACS). We aim to identify respiratory polygraphy (RP) patterns that contribute to identifying the risk of recurrent cardiovascular events in patients with ACS.

**Methods:**

*Post hoc* analysis of the ISAACC study, including 723 patients admitted for a first ACS (NCT01335087) in which RP was performed. To identify specific RP patterns, a principal component analysis (PCA) was performed using six RP parameters: AHI, oxygen desaturation index, mean and minimum oxygen saturation (SaO_2_), average duration of events and percentage of time with SaO_2_ < 90%. An independent HypnoLaus population-based cohort was used to validate the RP components.

**Results:**

From the ISAACC study, PCA showed that two RP components accounted for 70% of the variance in the RP data. These components were validated in the HypnoLaus cohort, with two similar RP components that explained 71.3% of the variance in the RP data. The first component (component 1) was mainly characterized by low mean SaO_2_ and obstructive respiratory events with severe desaturation, and the second component (component 2) was characterized by high mean SaO_2_ and long-duration obstructive respiratory events without severe desaturation. In the ISAACC cohort, component 2 was associated with an increased risk of recurrent cardiovascular events in the third tertile with an adjusted hazard ratio (95% CI) of 2.44 (1.07 to 5.56; *p*-value = 0.03) compared to first tertile. For component 1, no significant association was found for the risk of recurrent cardiovascular events.

**Conclusion:**

A RP component, mainly characterized by intermittent hypoxemia, is associated with a high risk of recurrent cardiovascular events in patients without previous CVD who have suffered a first ACS.

## Introduction

Cardiovascular diseases (CVDs) are one of the leading causes of death worldwide, and more than half are consequences of coronary heart disease, which is often manifested for the first time as acute coronary syndrome (ACS). Ranging from unstable angina to myocardial infarction, ACS is the main cause of morbidity and mortality worldwide (1, 2).

Obstructive sleep apnea (OSA) is a prevalent and chronic disease that affects more than 20% of the adult population (3) and is caused by the collapse of the upper airway during sleep, which leads to transient asphyxia. These events lead to brain arousal, intermittent hypoxemia, poor quality of life and metabolic disturbances. OSA is considered an important public health problem and is largely associated with cardiovascular outcomes (4, 5). In fact, basic, epidemiological and clinical research suggests a role of OSA in the initiation and/or progression of several CVDs (6). Observational studies have suggested that OSA is an independent risk factor for cardiovascular morbidity and mortality (7). However, there is a lack of a dose-response relationship between the severity of the disease [expressed by the apnea hypopnea index (AHI)] and cardiovascular risk, and some studies did not find an association between OSA and cardiovascular risk (8, 9). In the ISAAC study, a randomized controlled trial to evaluate the effect of intervention with continuous positive airway pressure (CPAP) treatment on the risk of recurrence of cardiovascular event in patients with ACS, OSA was not associated with a worse cardiovascular risk prognosis in patients with ACS (8). A *post hoc* study from the ISAACC study reported that the effect of OSA is heterogeneous and it was identified a specific endotype of patients with ACS where OSA was associated with an increased risk of recurrent cardiovascular events. The results of this *post hoc* study suggested that patients with OSA admitted by a first ACS without previous CVD present an increased risk of recurrent cardiovascular events (9). ISAACC study and others intervention trials failed to find a positive effect of CPAP treatment in the reduction of cardiovascular events (8, 10, 11) on the risk of cardiovascular recurrence.

Recent study (12) reported that CVD mortality was not associated with the AHI when it was assessed as an independent predictor. In contrast, they found that the hypoxic burden, an estimation of the depth and duration of respiratory-related desaturation, is an alternative metric associated with an increased risk of CVD mortality. Some authors argue that the AHI may not be a real marker of clinical disease and encourage further investigation of underlying pathophysiological and clinical phenotypes that are not captured by this measure (13). The AHI does not provide information about apnea depth and length and is a simplistic measure that has many downsides and could result in inadequate capture of the severity of the disease. Respiratory polygraphy (RP) allows the evaluation of a large number of physiological signals associated with respiratory disorders during sleep. Polygraphic recordings include at least oronasal flow, thoracoabdominal movements, heart rate, and oxygen saturation. Dimensionality reduction technique allows the transformation of data from a high-dimensional space into a low-dimensional space so that the low-dimensional representation retains some meaningful properties of the original data. The dimensionality reduction analysis of the signals of RP could contribute to the construction of models that would relate OSA to cardiovascular risk.

Available evidence supports that the indicators of cardiovascular risk in patients with OSA, mainly based on AHI, are insufficient. In the present study, we aimed to evaluate the RP patterns that could contribute to identifying the risk of recurrent cardiovascular events in patients with a first ACS and without previous CVD.

## Materials and Methods

### Study Design

**FIGURE 1:**
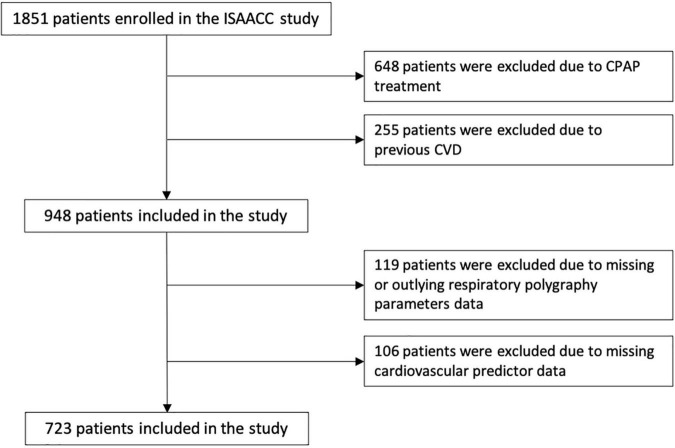
Flowchart of study. CPAP, continuous positive airway pressure; CVD, cardiovascular disease.

This is an ancillary study of the ISAACC (Impact of Sleep Apnea syndrome in the evolution of ACS. Effect of intervention with CPAP) study, a multicenter, open-label, parallel, prospective, randomized, controlled trial (registered trial NCT01335087). Full details of the ISAACC study’s methods, including inclusion and exclusion criteria, have been previously reported (14). Briefly, eligible patients were ≥18 years old, had been admitted for ACS to coronary care units or cardiology hospitalization ward at 15 hospitals in Spain and had an Epworth Sleepiness Scale score of ≤10 (i.e., patients without excessive daytime sleepiness or non-sleepy). Eligible patients underwent RP (Embletta, ResMed, Bella Vista, NSW, Australia) during the first 24–72 h after admission to evaluate the presence of OSA (14). For the current *post hoc* study, we analyzed the data from 723 ISAACC patients with a first ACS and without CVD (Figure 1) and in whom a deleterious effect of OSA was observed (13).

Acute coronary syndrome was defined as the acute presentation of coronary disease with or without ST elevation infarction, unstable angina, or type 1 myocardial infarction.

The ethics committee of each participating center approved the study (approval number in the coordinating center: 2010-852) and patients provided written informed consent.

The RP components in the ISAACC cohort was externally validated in the HypnoLaus population-based cohort (3), a nested-study of CoLaus/PsyCoLaus (15, 16). This study was designed to assess the prevalence of sleep-disordered breathing in a general population aged 35–75 years who were selected at random and were enrolled in the study.

### Procedures and Outcomes

#### ISAACC Cohort

Questionnaires to record demographic and anthropometric characteristics, medical history and usual pharmacological treatment were administered the day before the sleep study along with questionnaires associated with quality of life (EuroQol-5D questionnaire) and the degree of daytime sleepiness (Epworth Sleepiness Scale test). All patients were evaluated at baseline and 1 month, 3 months, 6 months, 12 months, 18 months, 24 months, 30 months, 36 months, and annually thereafter. All patients were monitored and followed up for a minimum of 1 year. At each visit, sociodemographic and anthropometric variables previously related to increased cardiovascular risk were recorded. Each follow-up visit included assessments of the rate of a composite of cardiovascular events [cardiovascular death or non-fatal events (acute myocardial infarction, non-fatal stroke, hospital admission for heart failure and new hospitalizations for unstable angina or transient ischemic attack)].

For the present study, we explored RP parameters to identify specific components, including the AHI, oxygen desaturation index (ODI, >4%), mean and minimum oxygen saturation (SaO_2_), average duration of events and percentage of time with SaO_2_ < 90%. We explored the contribution of RP components to the risk of recurrent cardiovascular events.

The cardiovascular risk variables explored were age, sex, current cigarette smoking, current alcohol consumption, obesity, hypertension, previous cerebrovascular disease, diabetes mellitus, and dyslipidemia (low-density lipoprotein cholesterol ≥100 mg/dL or the use of lipid-lowering drugs). Moreover, on the basis of cardiovascular risk prediction models (17, 18), we explored the following other cardiovascular variables: antihypertensive drugs, antiplatelet, and antithrombotic drugs, heart rate, systolic blood pressure, initial serum creatinine, stents implanted, no in-hospital percutaneous coronary intervention troponin and mean SaO_2_.

#### HypnoLaus Cohort

Participants underwent full polysomnography at home. Details of the sampling and procedure methodologies have been described elsewhere (3, 15). For the current study, we analyzed the data from 1941 HypnoLaus patients. The ethics committee of the University of Lausanne approved the CoLaus/PsyCoLaus cohort study and the HypnoLaus Sleep Cohort study. We obtained written informed consent from all participants.

### Statistical Analysis

The main characteristics were described using medians (25th percentile and 75th percentile) or percentages. The *P* value for trend was evaluated using Spearman’s rank correlation coefficient when data were continuous and non-normally distributed and the chi-square test for trend when they were categorical (19).

Principal component analysis (PCA) was used to avoid collinearity between the RP parameters and to identify relational patterns that reflect sleep apnea patterns. Six RP standardized parameters previously mentioned were used in the PCA. RP parameters that were not normally distributed were log-transformed and then included in the PCA (ODI and AHI). The magnitude of the eigenvalues > 1 (20) and variance explained by each component were used to determine the number of principal components to select. The components are described by the correlations with each RP parameter included in the PCA. Selected components from the PCA were grouped into tertiles and included in the model to explore the association with the primary composite endpoint. Cox proportional regression models adjusted by the predictors mentioned in the previous section were used. Hazard ratios (HRs) and 95% confidence intervals (95% CIs) were reported. The proportional hazard assumption was checked in all models. Discrimination was assessed using the C-statistic (21).

Statistical analyses were conducted using R, version 3.6 (22). Two-sided *p*-values were reported, and statistical significance was set at 0.05.

## Results

### Patient Characteristics

#### ISAACC Cohort

From a total of 1,851 patients with ACS enrolled in the ISAACC cohort, we included 948 patients without CPAP treatment and without previous CVD. Finally, 723 patients with available data for the variables explored were included in the present study (Figure 1). The median age of the patients was 58 years, and 81.6% were males (Table 1). The patients were followed for a median of 3.33 (interquartile range: 3.72) years. Excluded patients had sociodemographic and clinical characteristics similar to those of the patients included in the analysis (Supplementary Table 1). A total of 11.5% of the patients had recurrent cardiovascular events during follow-up (Supplementary Table 2).

**TABLE 1:** Baseline characteristics of patients from the ISAACC cohort.

	All	No recurrent CVE	Recurrent CVE	*P* value

	**(*N* = 723)**	**(*N* = 640)**	**(*N* = 83)**	
**Sex**				0.203
Female	133 (18.4%)	113 (17.7%)	20 (24.1%)	
Male	590 (81.6%)	527 (82.3%)	63 (75.9%)	
**Anthropometric measures**				
Age, years	58.0 [51.0;66.0]	58.0 [51.0;66.0]	58.0 [52.5;66.5]	0.426
Body mass index, kg⋅m^–2^	28.0 [25.1;30.8]	27.8 [25.0;30.5]	28.9 [25.6;31.3]	0.070
Waist-hip ratio	0.98 [0.94;1.02]	0.98 [0.94;1.02]	0.99 [0.95;1.03]	0.435
Neck circumference, cm	40.0 [38.0;43.0]	40.0 [38.0;43.0]	40.0 [38.0;42.0]	0.662
**Lifestyle risk factors**				
Smoking				0.838
Never	189 (26.1%)	166 (25.9%)	23 (27.7%)	
Former	166 (23.0%)	149 (23.3%)	17 (20.5%)	
Current	368 (50.9%)	325 (50.8%)	43 (51.8%)	
Drinking				0.168
No	534 (73.9%)	467 (73.0%)	67 (80.7%)	
Yes	189 (26.1%)	173 (27.0%)	16 (19.3%)	
**Sleep parameters**				
AHI, events per h	16.5 [7.00;33.3]	15.9 [6.70;33.0]	20.2 [11.1;37.1]	**0.016**
ODI > 4%, per h	14.0 [5.25;29.9]	13.9 [5.00;29.3]	16.7 [8.75;34.9]	0.061
Mean SaO_2_,%	93.1 [92.0;94.4]	93.1 [92.0;94.3]	93.3 [92.0;94.5]	0.785
Minimum SaO_2_,%	86.0 [81.0;88.0]	86.0 [81.0;88.0]	86.0 [82.0;89.0]	0.351
Time with SaO_2_ < 90%, %	1.00 [0.10;7.25]	1.05 [0.10;7.35]	0.60 [0.00;6.80]	0.512
Average duration of events, sec	20.0 [17.0;23.0]	20.0 [17.0;23.0]	19.0 [17.0;22.5]	0.464
Epworth sleepiness scale	5.00 [3.00;7.00]	5.00 [3.00;7.00]	5.00 [3.00;7.00]	0.152
**Medical history**				
Hypertension	325 (45.0%)	278 (43.4%)	47 (56.6%)	**0.031**
Diabetes mellitus	141 (19.5%)	112 (17.5%)	29 (34.9%)	**<0.001**
Dyslipidemia	596 (82.4%)	530 (82.8%)	66 (79.5%)	0.556
Cerebrovascular disease	16 (2.21%)	12 (1.88%)	4 (4.82%)	0.100
Chronic pneumopathy	35 (4.84%)	32 (5.00%)	3 (3.61%)	0.787
Neurological disease	31 (4.29%)	26 (4.06%)	5 (6.02%)	0.387
**Severity of ACS**				0.331
Mild	653 (90.3%)	581 (90.8%)	72 (86.7%)	
Severe	70 (9.68%)	59 (9.22%)	11 (13.3%)	
**Medication**				
Antihypertensive drug	293 (40.5%)	251 (39.2%)	42 (50.6%)	0.062
Lipid-lowering drug	188 (26.0%)	161 (25.2%)	27 (32.5%)	0.191
Antidiabetic oral medication	113 (15.6%)	87 (13.6%)	26 (31.3%)	**<0.001**
Insulin	26 (3.60%)	21 (3.28%)	5 (6.02%)	0.207
Antiplatelet and antithrombotic drugs	69 (9.54%)	45 (7.03%)	24 (28.9%)	**<0.001**
**Cardiovascular variables**				
Heart rate, bpm	70.0 [63.5;80.0]	70.0 [63.0;80.0]	73.0 [65.0;80.5]	0.125
Systolic blood pressure, mm Hg	120 [110;133]	120 [110;133]	120 [110;130]	0.608
Creatinine, mg/dL	0.85 [0.73;0.99]	0.84 [0.73;0.98]	0.88 [0.74;1.02]	0.131
Stents implanted	652 (90.2%)	575 (89.8%)	77 (92.8%)	0.518
**Peak troponin, quartiles**				0.070
Quartile 1	130 (18.0%)	107 (16.7%)	23 (27.7%)	
Quartile 2	191 (26.4%)	171 (26.7%)	20 (24.1%)	
Quartile 3	203 (28.1%)	186 (29.1%)	17 (20.5%)	
Quartile 4	199 (27.5%)	176 (27.5%)	23 (27.7%)	
**Cardiovascular event type**				0.818
Non-Q-wave AMI	299 (41.4%)	264 (41.2%)	35 (42.2%)	
Unstable angina	37 (5.12%)	32 (5.00%)	5 (6.02%)	
Q-wave AMI	387 (53.5%)	344 (53.8%)	43 (51.8%)	

*Data are presented as the n (%) or the median [25th percentile; 75th percentile]. CVE, cardiovascular event; AHI, apnea-hypopnea index; ODI, oxygen desaturation index; SaO_2_, oxygen saturation; ACS, acute coronary syndrome; bpm, beats per minute; and AMI, acute myocardial infarction. Significant p values (p < 0.05) are presented in bold.*

#### HypnoLaus Cohort

The external validation cohort was 2,168 individuals from the HypnoLaus population-based cohort. Finally, 1941 individuals were selected without previous CVD and available data. The median age was 56.9 years, 47% were males, and the median AHI was 9.8 events per hour (Supplementary Table 3). The mean time of follow-up (or CV event) was 4.25 (standard deviation: 1.77) years.

### Identification of Respiratory Polygraphy Components

**FIGURE 2:**
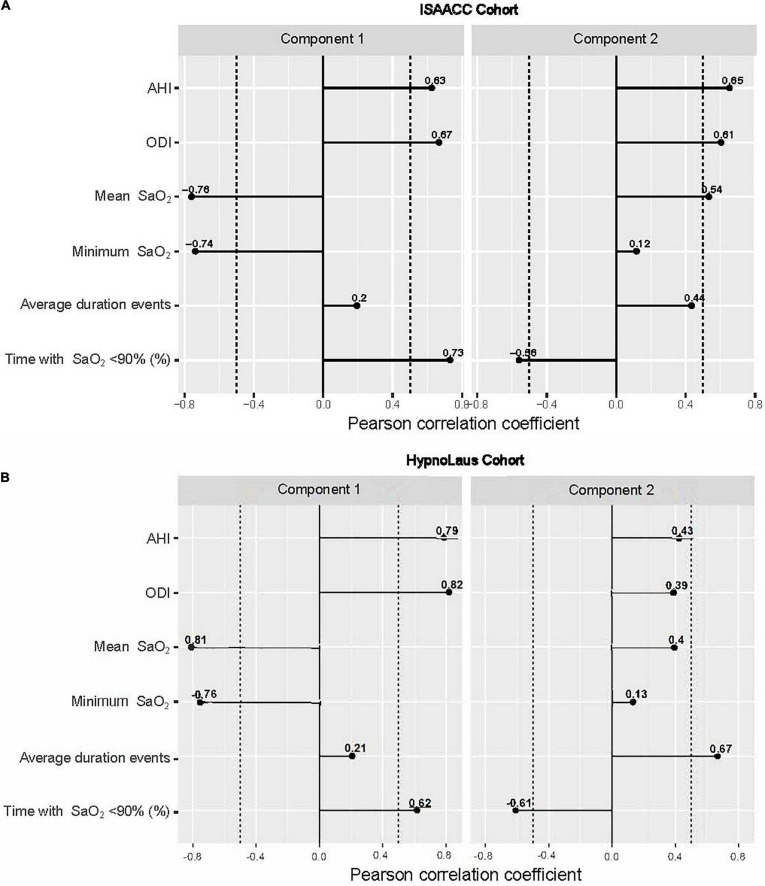
Correlations between respiratory polygraphy parameters and components derived from the PCA. **(A)** In the ISAACC cohort, these two components, component 1 and component 2, accounted for 43 and 26% of the total variance, respectively. **(B)** In HypnoLaus, these two components, component 1 and component 2, accounted for 49.2 and 22.1% of the total variance, respectively. AHI, apnea-hypopnea index; ODI, oxygen desaturation index; and SaO_2_, oxygen saturation. Dashed lines represent –0.5 and 0.5 correlations.

Principal component analysis was performed using the six RP parameters: AHI, ODI, mean and minimum SaO_2_, average duration of events and percentage of time with SaO_2_ <90%. The first two components were selected to characterize the RP patterns. In the ISAACC study, these two components, which contributed significantly to explaining the relationships among the six RP parameters (eigenvalues >1), accounted for 70% of the variability of the RP data (Supplementary Table 4). The first component (Component 1) was highly correlated with the AHI, ODI and percentage of time with SaO_2_ <90% and inversely correlated with the mean and minimum SaO_2_. Component 1 accounted for 42.2% of the variability of the RP data. The second component (Component 2) was highly correlated with the AHI, ODI, mean SaO_2_, and average duration of events and inversely correlated with the percentage of time with SaO_2_ <90% (Figure 2A). Component 2 accounted for 26.6% of the variability of the RP data. Whereas the mean and minimum SaO_2_ were negatively correlated in component 1, in the component 2 mean SaO_2_ was positively correlated. Additionally, the correlation of duration obstructive respiratory events was higher in component 2. Table 2 shows the description of the RP parameters by tertiles of each RP component identified and selected (components 1 and 2).

**TABLE 2:** Description of the respiratory polygraphy parameters by tertiles of the components derived from the PCA in the ISAACC cohort.

	Component 1			Component 2				
	1st tertile	2nd tertile	3rd tertile	*p* for trend	1st tertile	2nd tertile	3rd tertile	*p* for trend
	(*N* = 239)	(*N* = 238)	(*N* = 246)		(*N* = 239)	(*N* = 238)	(*N* = 246)	
**RP parameters**								
AHI, events per h	6 [8.6]	18 [18]	35.2 [31.5]	**<0.001**	5.3[11.7]	15.8 [17.2]	32.8 [26.3]	**<0.001**
ODI > 4%, per h	4.2 [5.9]	15.1 [13.8]	32.9 [30.9]	**<0.001**	4 [11.6]	13 [16.7]	27.6 [25.4]	**<0.001**
Mean SaO_2_,%	94.3 [1.9]	93.2 [1.7]	91.8 [2.9]	**<0.001**	92.2 [3.7]	93 [2]	94 [2]	**<0.001**
Minimum SaO_2_,%	89 [4]	86 [4]	80 [8.8]	**<0.001**	85 [8]	86 [6]	85.5 [6]	0.312
Average duration of events, sec	19 [7]	20 [6]	20 [7.8]	**<0.001**	17 [6]	20 [6]	22 [8]	**<0.001**
Time with SaO_2_ < 90%,%	0 [0.3]	0.8 [2.2]	13 [29.3]	**<0.001**	3.1[33.4]	0.8 [5.1]	0.9 [3.8]	**<0.001**

*Data are presented as the median [IQR (interquartile range)]. PCA, principal component analysis; RP, respiratory polygraphy; AHI, apnea-hypopnea index; ODI, oxygen desaturation index; and SaO_2_, oxygen saturation. The p value for trend was calculated using Spearman’s rank correlation coefficient. Significant p values (p < 0.05) are presented in bold.*

From the validation, the PCA was repeated in the HypnoLaus cohort using the same six RP parameters explored in the ISAACC cohort, and the first two components explained 71.3% of the variance of the RP data (Supplementary Table 5). Both components presented a high degree of similarity to those identified in the ISAACC cohort (Figure 2B). The RP parameters were described by tertiles of the selected component for the HypnoLaus cohort (Supplementary Table 6).

### Respiratory Polygraphy Component Distribution

ISAACC patients were characterized using sociodemographic and clinical characteristics by tertiles of the two RP components (Supplementary Table 7). Patients with high adherence in component 1 had more comorbidities, as the third tertile showed a greater proportion of patients with obesity, hypertension and diabetes. Similar characteristics were shown by tertiles of component 2.

In the same way, HypnoLaus subjects were described using sociodemographic and clinical characteristics by tertiles of the two RP components (Supplementary Table 8). Subjects with high adherence in component 1 had more comorbidities, as third tertile showed a greater proportion of subjects with hypertension, diabetes, dyslipidemia and a higher body mass index (BMI). In component 2, the third tertile showed a greater proportion of subjects with hypertension, a lower proportion of subjects with dyslipidemia and no differences regarding BMI and diabetes.

### Relationship Between Respiratory Polygraphy Components and Recurrent Cardiovascular Risk

In the present study, from the ISAACC study, we explored specific RP parameters and components (Supplementary Table 9) in the subgroup of patients admitted by a first ACS and without previous CVD (*n* = 723).

In the subgroup of patients without previous ACS, OSA severity (measured by AHI) showed a trend toward statistical association [HR (95% CI) of 1.62 (0.98–2.66)] with an increased risk of recurrent cardiovascular events (Supplementary Table 10).

**FIGURE 3:**
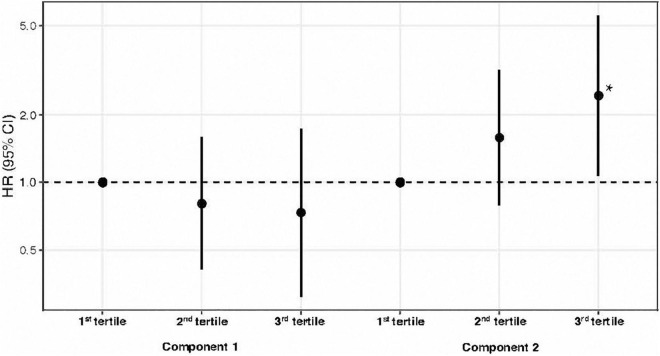
Hazard ratio for recurrent cardiovascular events by tertiles of respiratory polygraphy components in the ISAACC cohort. Dots are hazard ratios, and lines represent 95% confidence intervals. Hazard ratios were adjusted for age, sex, smoking, alcohol consumption, obesity, hypertension, stroke, diabetes, the severity of ACS, dyslipidemia, antihypertensive drugs, antiplatelet and antithrombotic drugs, heart rate, systolic blood pressure, creatinine, stents implanted, cardiovascular event type, troponin peak, and mean SaO_2_. HR (95% CI), hazard ratio (95% confidence interval); ACS, acute coronary syndrome. *Significant *p* values (*p* < 0.05).

In the analysis of RP components and the risk of recurrent cardiovascular events, it was observed that component 2 showed a significant independent association with the risk of recurrent cardiovascular events in patients with the highest adherence (3rd tertile) of component 2 with an adjusted HR (95% CI) of 2.44 (1.07 to 5.56). For component 1, no significant contribution was found to the risk of recurrent cardiovascular events (Table 3 and Figure 3). The discriminatory power of the models showed a C-statistic for the risk of recurrent cardiovascular events of 72.3% for patients without previous CVD. Similar results were found exploring components from PCA as continuous [HR (95%CI): for Component 2: 1.38 (0.99–1.94) and for Component 1: 0.82 (0.6–1.13)].

**TABLE 3:** Cox proportional hazard model for the primary composite endpoint in the ISAACC cohort.

	ALL (*N* = 723)		
	HR* (95% CI)	*p* value	*p* for trend
**Component 1**			
1st tertile	1		0.49
2nd tertile	0.81 (0.41–1.59)	0.544	
3rd tertile	0.74 (0.31–1.73)	0.481	
**Component 2**			
1st tertile	1		**0.03**
2nd tertile	1.58 (0.79–3.17)	0.194	
3rd tertile	2.44 (1.07–5.56)	**0.034**	

*HR (95% CI), hazard ratio (95% confidence interval). All variables included in the model met the assumptions for proportional hazards. The p value for trend was computed by treating component variables coded as numbers in the model. Significant p values (p < 0.05) are presented in bold.*

*Adjusted for age, sex, smoking, alcohol consumption, obesity, hypertension, stroke, diabetes, the severity of ACS, dyslipidemia, antihypertensive drugs, antiplatelet and antithrombotic drugs, heart rate, systolic blood pressure, creatinine, stents implanted, cardiovascular event type, troponin peak, and mean SaO_2_.

In the HypnoLaus cohort the risk of recurrent cardiovascular event in patients with first ACS could not be assessed due to its population-based design.

## Discussion

In the present study, we explored the relationship between specific components of sleep-disordered breathing and the risk of recurrent cardiovascular events in a cohort of patients with a first ACS and without previous CVD. Recently, it has been reported that patients with OSA admitted by a first ACS without previous CVD present an increased risk of recurrent cardiovascular events (13). The results of this study show an association between a specific polygraphy component and the risk of recurrent cardiovascular events. In the ISAACC population, we report that a polygraphy component mainly characterized by intermittent hypoxemia (component 2) increases the risk of recurrent cardiovascular events in patients with ACS and without previous CVD. Based on previously described cardiovascular risk variables, the C-statistic for the risk of recurrent cardiovascular events was 72% for both phenotypes of patients, similar to other studies (23). Remarkably, the RP components identified in the ISAACC cohort were also identified in the HypnoLaus population-based cohort.

Currently, OSA severity is characterized by the AHI, which captures only one aspect of this heterogeneous disease. The ability to dissect OSA heterogeneity using additional features is necessary for incorporating personalized approaches in disease management. In our study, we observed that the effect of OSA severity, assessed only by the AHI, showed a trend toward a statistical association (with an HR of 1.62) with the risk of recurrent cardiovascular events in the group of patients without previous CVD. When considering the RP components, we observed an HR of 2.44 for component 2, indicating an increase in the risk of recurrent cardiovascular events. Based on the results, we suggest that the analysis of other parameters from RP could contribute to the determination of RP components and their relationship with the development of recurrent cardiovascular events in patients who have suffered an ACS.

The ISAACC study (8) and others (12) have failed to demonstrate the relationship between the AHI and an increased risk of recurrent cardiovascular events. Previous studies have suggested that other components from sleep studies could contribute to characterizing the underlying pathology of OSA and its association with the risk of CVD. Azarbarzin et al. (12) recently reported that CVD mortality was not associated with the AHI when it was assessed as an independent predictor. In contrast, they found that the hypoxic burden, an estimation of the depth and duration of respiratory-related desaturation, is an alternative metric associated with an increased risk of CVD mortality. Nevertheless, the authors of that study admit that this measure does not fully characterize the components of hypoxic stress because it does not distinguish short and deep desaturations from long and shallow desaturations. In fact, in our study, we found that the component associated with recurrent cardiovascular events was component 2. The highest adherence to this component (3rd tertile), showing the highest risk of recurrent cardiovascular events, was characterized by higher AHI, higher mean SaO_2_ and longer event duration. This tertile would suggest an intermittent hypoxemia component. In contrast, the lowest adherence to this component was characterized by lower AHI, ODI and mean SaO_2_, which would suggest a sustained hypoxemia component.

The results of the present study suggest that from available variables from RP, it is possible to improve the evaluation of the risk of recurrent cardiovascular events with an HR of 1.61 exclusively with AHI to 2.44 considering additional variables. The patients with the highest adherence to component 2 (3rd tertile), which represents an intermittent hypoxemia component, strengthen the hypothesis that OSA exerts a risk in specific subgroups of patients with ACS. In contrast, the patients with the lowest adherence to this component, characterized by sustained hypoxemia, would have a lower risk of recurrence of a cardiovascular event. Notably, the same RP components were found in the HypnoLaus cohort suggesting that these components are not specific to the ISAACC population. However, it was not possible to estimate the predictive nature of the identified RP components for recurrent cardiovascular events in this cohort. Since, the low number of available cardiovascular events due to its population-based design.

The existence of specific RP components that could be associated with detrimental effects in specific phenotypes of patients with ACS invites us to reconsider the design strategies of new interventional studies to demonstrate the possible beneficial effect of CPAP treatment on secondary CVD prevention. These preliminary results raise the possibility that, in studies such as the ISAACC study (8), no beneficial effect was seen because of the inclusion of patients with different phenotypes of ACS where OSA could have different effects.

The strengths of our study include its multicenter design with a large population of patients with diagnoses of ACS and OSA. In this study, all participating centers from the ISAACC study used the same methodology, and the sleep study was performed with the same polygraph model. In addition, the RP components identified in the ISAACC cohort were validated in the HypnoLaus population-based cohort. The study presents some limitations that deserve mention. First, to identify specific RP components, a PCA analysis was performed using six RP parameters, including the AHI, ODI, mean and minimum SaO_2_, average duration of events and percentage of time with SaO_2_ < 90%. The usefulness of other variables such as the mean respiratory rate as a predictor of cardiovascular recurrence was previously reported. This variable was not available in the ISAACC study, and future studies exploring the contribution of this specific variable as a predictor of cardiovascular outcomes should be performed. Second, the results of this study indicate the possibility of identifying RP components associated with different risks of recurrent cardiovascular events in patients who have suffered a first ACS and without previous CVD. However, these exploratory results should be validated in future independent cohorts. Third, in our study, we determined RP components using RP parameters that do not include information about sleep phases. Some studies have suggested that rapid eye movement-related respiratory disorders could be informative regarding cardiovascular risk associations with respiratory sleep disorders (24). The contribution of information about sleep phases should be explored in further studies. Finally, the results from the present study may not be extrapolated to a population different from that of patients admitted to the hospital for ACS. This fact makes it necessary to specifically identify phenotypes in patients with OSA who are attended in different clinical settings.

As a conclusion, in patients who have suffered a first ACS and without previous CVD, there exists a specific RP component, mainly characterized by intermittent hypoxemia, that is associated with a greater risk of recurrent cardiovascular events. These first exploratory results must be confirmed in future studies, which will evaluate the effect of RP components of specific ACS populations and the possible beneficial effect of CPAP treatment for patients with a first ACS and without previous CVD, in whom a deleterious effect of this RP component has been found.

## Data Availability Statement

The original contributions presented in the study are included in the article/Supplementary Material, further inquiries can be directed to the corresponding authors.

## Ethics Statement

The ethics committee of each participating center approved the study (approval number in the coordinating center: 2010-852) and patients provided written informed consent. The patients/participants provided their written informed consent to participate in this study.

## Members of the Spanish Sleep Network

Laura Abad, Aida Munϸoz, Elisabet Zamora, Ignacio Vicente, Sandra Ingleìs, Carlos Egea, Jaime Marcos, Almudena Fernaìndez, Valentin Cabriada-Nuño, Sonia Castro, Leyre Serrano, Marina Floreìs, Anna Mas, Maricel Arboneìs, Silvia Ortega, Alicia Martiìn, Jose Miguel Romaìn-Saìnchez, Ma Isabel Valiente-Diaz, Ma Esther Viejo-Ayuso, Concepcioìn Rodriìguez-Garciìa, Laura Vigil, Enriqueta Ramiìrez, Mariìa Pinϸar, Elisabet Martiìnez, Blanca Barriuso, Jaime Corral, Francisco Javier Goìmez de Terreros Caro, Antonia Barceloì, Paloma Gimeìnez, Ana Ma Fortuna, Patricia Penñacoba, Abel Jesuìs Martiìnez-Garciìa, Sergio Garciìa-Castillo, Lara Navas, Onintza Garmendia, Joseì Sancho, Salvador Perelloì, Gemma Rubinoìs, and Rocío Gallego.

## Author Contributions

AZ, GS, AS-d-l-T, EG-L, IB, FB, RH, and MS-d-l-T contributed to the study concept and design. GS, AS-d-l-T, GT, JD, JA, JD-C, AU, OMe, MJM, EO-C, JFM, MDP, MM, RC, JMM, EC, OMí, LP, AC, DM, RM, and RH contributed to the data acquisition. AZ, GS, AS-d-l-T, EG-L, IB, GT, FB, RH, and MS-d-l-T contributed to the data analysis and interpretation. All authors contributed to the drafting of the manuscript, critically revised the manuscript for important intellectual content and approved the final version. MS-d-l-T is the guarantor of the manuscript.

## Conflict of Interest

The authors declare that the research was conducted in the absence of any commercial or financial relationships that could be construed as a potential conflict of interest.

## Publisher’s Note

All claims expressed in this article are solely those of the authors and do not necessarily represent those of their affiliated organizations, or those of the publisher, the editors and the reviewers. Any product that may be evaluated in this article, or claim that may be made by its manufacturer, is not guaranteed or endorsed by the publisher.

## Abbreviations

ACS: acute coronary syndrome
AHI: apnea-hypopnea index
CI: confidence interval
CPAP: continuous positive airway pressure
CVD: cardiovascular disease
HR: hazard ratio
IQR: interquartile rang
ODI: oxygen desaturation index
OSA: obstructive sleep apnea
PCA: principal component analysis
SaO_2_: oxygen saturation.
