# Supplementary material for: Respiratory Polygraphy Patterns and Risk of Recurrent Cardiovascular Events in Patients With Acute Coronary Syndrome
Source: Front Med (Lausanne). 2022 Jun 27;9:870906. doi: 10.3389/fmed.2022.870906 (PMC9271863; doi:10.3389/fmed.2022.870906)
Supplement: Supplementary file 1 [file Data_Sheet_2.DOCX]

Supplementary Material

# Supplementary methods

## *Study design*

In this study, in the ISAACC cohort, the exclusion criteria were as follows: previous treatment with CPAP; a psychological or physical inability to complete questionnaires; the presence of any previously diagnosed sleep disorder; >50% central apnea or the presence of Cheyne–Stokes respiration; daytime sleepiness (Epworth Sleepiness Scale score >10); chronic diseases (e.g., neoplasms, renal insufficiency [glomerular filtration rate <15 mL·min−1·1·73 m^-2^], severe chronic obstructive pulmonary disorder [a forced expiratory volume in 1 s <50%], chronic depression or other limiting chronic diseases); a history of a medical condition that could interfere with the study objectives; any conditions, cardiovascular or otherwise, that could reduce life expectancy to <1 year; or cardiogenic shock.

## *Procedures and outcomes*

In the ISAACC study, questionnaires to record demographic and anthropometric characteristics, medical history and usual pharmacological treatment were administered. Eligible patients underwent respiratory polygraphy (Embletta; ResMed, Bella Vista, NSW, Australia) during the first 24–72 h after admission to the hospital for OSA diagnoses according to the national guidelines(1). Apnea was defined as an absence of airflow lasting 10 s or more. Hypopnea was defined as a reduction in airflow lasting 10 s or more associated with oxygen desaturation. Oxygen desaturation was defined as a decrease in the arterial SaO_2_ of more than 4%. The AHI was defined as the number of episodes of apnea and hypopnea per hour of recording. Respiratory polygraphy studies were performed without supplemental oxygen. From respiratory polygraphy, the following variables were obtained: AHI, ODI, mean SaO_2_, minimum SaO_2_, total time with SaO_2_ <90%, percentage of time with SaO_2_ <90, percentage of obstructive+mixed events, average duration of events, and % supine position.

All the patients from the ISAACC study were monitored and followed up for a minimum of one year. At each visit, sociodemographic and anthropometric variables previously determined to be related to increased cardiovascular risk were recorded. Each follow-up visit included assessments of the occurrence of a composite of cardiovascular events (cardiovascular death or nonfatal events [acute myocardial infarction, nonfatal stroke, hospital admission for heart failure, and new hospitalizations for unstable angina or transient ischemic attack]). The primary composite endpoint of the ISAACC study was the occurrence of one of the composite cardiovascular events.

# Supplementary Tables

## Table E1: Baseline characteristics of the participants included versus excluded from the ISAACC cohort.

|  | **Excluded** | **Included** |
| --- | --- | --- |
|  | **(N=225)** | **(N=723)** |
| **Cardiovascular risk predictors** |  |  |
| Age, years | 56.0 [50.0;65.0] | 58.0 [51.0;66.0] |
| Sex |  |  |
| Female | 32 (14.2%) | 133 (18.4%) |
| Male | 193 (85.8%) | 590 (81.6%) |
| Body Mass Index, kg·m^-2^ | 27.5 [25.4;30.1] | 28.0 [25.1;30.8] |
| Smoking |  |  |
| Never | 52 (23.1%) | 189 (26.1%) |
| Former | 53 (23.6%) | 166 (23.0%) |
| Current | 120 (53.3%) | 368 (50.9%) |
| Drinking |  |  |
| No | 147 (75.4%) | 534 (73.9%) |
| Yes | 48 (24.6%) | 189 (26.1%) |
| Hypertension | 100 (44.4%) | 325 (45.0%) |
| Cerebrovascular disease | 5 (2.23%) | 16 (2.21%) |
| Diabetes mellitus | 39 (17.3%) | 141 (19.5%) |
| Severity of ACS |  |  |
| Mild | 177 (86.8%) | 653 (90.3%) |
| Severe | 27 (13.2%) | 70 (9.68%) |
| Dyslipidemia | 173 (76.9%) | 596 (82.4%) |
| **Sleep parameters** |  |  |
| AHI, events per h | 10.6 [4.20;24.8] | 16.5 [7.00;33.3] |
| ODI > 4%, per h | 10.5 [3.12;24.9] | 14.0 [5.25;29.9] |
| Mean SaO2, % | 93.4 [92.0;94.5] | 93.1 [92.0;94.4] |
| Minimum SaO_2_, % | 86.0 [82.0;90.0] | 86.0 [81.0;88.0] |
| Average duration of events, sec | 19.0 [16.0;24.0] | 20.0 [17.0;23.0] |
| Time with SaO_2_ < 90%, % | 1.60 [0.10;9.03] | 1.00 [0.10;7.25] |
| Epworth Sleepiness Scale | 5.00 [3.00;7.00] | 5.00 [3.00;7.00] |
| **Primary composite endpoint** |  |  |
| No | 199 (88.4%) | 640 (88.5%) |
| Yes | 26 (11.6%) | 83 (11.5%) |

The data are presented as the n (%) or median [25th percentile; 75th percentile]. AHI: apnea-hypopnea index; ODI: oxygen desaturation index; SaO_2_: oxygen saturation.

## Table E2. Cardiovascular outcomes during follow-up from the ISAACC cohort (N=83).

|  | **N (%)** |
| --- | --- |
| **Cardiovascular death** | 3 (3.6%) |
| **ACS** | 31 (37.3%) |
| **Stroke** | 2 (2.4%) |
| **Hospitalization: TIA** | 11 (13.3%) |
| **Hospitalization: unstable angina** | 36 (43.4%) |

TIA= transient ischemic attack, ACS=acute coronary syndrome

## Table E3: Baseline characteristics of subjects in the HypnoLaus cohort.

|  | **All** |
| --- | --- |
|  | **(N=1941)** |
| **Sex** |  |
| Female | 1028 (53.0%) |
| Male | 913 (47.0%) |
| **Anthropometric measures** |  |
| Age, years | 56.9 [49.1;68.1] |
| Body mass index, kg·m^-2^ | 25.6 [23.0;28.4] |
| Waist-hip ratio | 0.9 [0.9;1.0] |
| Neck circumference, cm | 36.5 [33.5;39.5] |
| **Lifestyle risk factors** |  |
| Smoking |  |
| Never | 807 (41.6%) |
| Former | 763 (39.3%) |
| Current | 349 (18.0%) |
| Weekly alcohol consumption, units | 4.0 [1.0;9.0] |
| **Sleep parameters** |  |
| AHI, events per h | 9.8 [4.1;20.4] |
| ODI > 4%, per h | 3.9 [1.1;9.9] |
| Mean SaO_2_, % | 94.3 [93.2;95.4] |
| Minimum SaO_2_, % | 87.0 [82.0;90.0] |
| Average duration of events, sec | 23.0 [19.5;27.2] |
| Time with SaO_2_ < 90%, % | 0.1 [0.0;1.8] |
| Epworth Sleepiness Scale | 6.0 [3.0;9.0] |
| **Medical history** |  |
| Hypertension | 759 (39.1%) |
| Diabetes mellitus | 173 (8.9%) |
| Dyslipidemia | 509 (26.2%) |
| **Medication** |  |
| Antihypertensive drug | 463 (23.9%) |
| Lipid-lowering drug | 366 (18.9%) |
| Antidiabetic oral medication | 89 (4.6%) |
| Insulin | 19 (1.0%) |
| **Cardiovascular variables** |  |
| New cardiovascular event* | 75 (3.9%) |
| Heart rate**, bpm | 62.3 [57.1;67.4] |
| Systolic blood pressure, mmHg | 124.0 [113.0;136.5] |

The data are presented as the n (%) or the median [25th percentile; 75th percentile]. AHI: Apnea-hypopnea index; ODI: oxygen desaturation index; SaO_2_: oxygen saturation; bpm: beats per minute.

*Cardiovascular events: any nonfatal CV event [stroke, heart attack, CABG or PCI] from PSG;FU2 [adjudicated]. **Average heart rate during sleep.

## Table E4: Correlations of respiratory polygraphy parameters with six components derived from the PCA in the ISAACC cohort.

| **Components** | **Comp 1** | **Comp 2** | **Comp 3** | **Comp 4** | **Comp 5** | **Comp 6** |
| --- | --- | --- | --- | --- | --- | --- |
| % variance | **42.2%** | **26.6%** | **15.5%** | **9.1%** | **3.7%** | **2.9%** |
| eigenvalue | 2.529 | 1.598 | 0.93 | 0.549 | 0.222 | 0.172 |
| **Respiratory polygraphy parameters** | |  |  |  |  |  |
| AHI, events per h | 0.628 | 0.654 | -0.252 | 0.132 | 0.262 | -0.170 |
| ODI > 4%, per h | 0.669 | 0.606 | -0.287 | -0.007 | -0.266 | 0.179 |
| Mean SaO_2_, % | -0.756 | 0.536 | -0.007 | -0.205 | 0.210 | 0.234 |
| Minimum SaO_2_, % | -0.735 | 0.119 | -0.240 | 0.620 | -0.055 | 0.016 |
| Average duration of events, sec | 0.196 | 0.437 | 0.852 | 0.209 | -0.027 | 0.016 |
| Time with SaO_2_ < 90%, % | 0.733 | -0.558 | 0.002 | 0.248 | 0.187 | 0.236 |

PCA: Principal component analysis; AHI: apnea-hypopnea index; ODI: oxygen desaturation index; SaO_2_: oxygen saturation.

## Table E5: Correlations of respiratory polygraphy parameters with six components derived from the PCA in the HypnoLaus study.

| **Components** | **Comp 1** | **Comp 2** | **Comp 3** | **Comp 4** | **Comp 5** | **Comp 6** |
| --- | --- | --- | --- | --- | --- | --- |
| % variance  eigenvalue | **49.2%** | **22.1%** | **13.2%** | **8.7%** | **4.2%** | **2.7%** |
|  | 2.95 | 1.32 | 0.79 | 0.52 | 0.25 | 0.16 |
| **Respiratory polygraphy parameters** | |  |  |  |  |  |
| AHI, events per h | 0.789 | 0.427 | -0.298 | 0.145 | 0.114 | -0.270 |
| ODI > 4%, per h | 0.821 | 0.391 | -0.261 | 0.133 | 0.019 | 0.295 |
| Mean SaO_2_, % | -0.811 | 0.395 | -0.107 | -0.162 | 0.383 | 0.048 |
| Minimum SaO_2_, % | -0.758 | 0.134 | -0.007 | 0.637 | -0.033 | 0.004 |
| Average duration of events, sec | 0.210 | 0.669 | 0.713 | -0.009 | -0.030 | -0.009 |
| Time with SaO_2_ < 90%, % | 0.618 | -0.608 | 0.336 | 0.212 | 0.301 | 0.023 |

PCA: principal component analysis; AHI: apnea-hypopnea index; ODI: oxygen desaturation index; SaO_2_: oxygen saturation.

## Table E6: Description of the respiratory polygraphy parameters by tertiles of the components derived from the PCA in the HypnoLaus study.

|  | **Component 1** | | | **Component 2** | | | | |
| --- | --- | --- | --- | --- | --- | --- | --- | --- |
|  | **1^st^ tertile** | **2^nd^ tertile** | **3^rd^ tertile** | **p for trend** | **1^st^ tertile** | **2^nd^ tertile** | **3^rd^ tertile** | **p for trend** |
|  | **[N=640]** | **[N=640]** | **[N=660]** |  | **[N=640]** | **[N=640]** | **[N=660]** |  |
| **Respiratory polygraphy parameters** | | |  |  |  |  |  |  |
| AHI, events per h | 2.9 [3.6] | 10.4 [8.3] | 25.3 [22.7] | **<0.001** | 3.9 [10.4] | 9.7 [12.8] | 15.7 [17.7] | **<0.001** |
| ODI > 4%, per h | 0.6 [1.1] | 4.1 [3.8] | 14.0 [15.6] | **<0.001** | 1.3 [6.1] | 3.8 [7.7] | 6.6 [10.3] | **<0.001** |
| Mean SaO_2_, % | 95.6 [1.4] | 94.3 [1.3] | 92.8 [1.9] | **<0.001** | 93.7 [3.7] | 94.2 [1.9] | 94.6 [1.5] | **<0.001** |
| Minimum SaO_2_, % | 91.0 [3.0] | 87.0 [4.0] | 82.0 [6.0] | **<0.001** | 86.0 [10.0] | 87.0 [6.0] | 86.0 [6.0] | 0.410 |
| Average duration of events, sec | 21.3 [7.4] | 24.0 [7.7] | 24.3 [7.6] | **<0.001** | 18.9 [4.9] | 22.3 [4.6] | 28.3 [6.1] | **<0.001** |
| Time with SaO_2_ < 90%, % | 0.0 [0.0] | 0.1 [0.4] | 3.7 [10.4] | **<0.001** | 0.1 [8.4] | 0.1 [1.3] | 0.2 [1.2] | 0.361 |

The data are presented as the median [IQR [interquartile range]]. Significant p values (p<0.05) are presented in bold. AHI: apnea-hypopnea index; ODI: oxygen desaturation index; SaO_2_: oxygen saturation. The p for trend was calculated using the Spearman correlation test.

## Table E7: Baseline characteristics by tertiles of respiratory polygraphy components in the ISAACC cohort.

|  | **Component 1** | | | | | | | **Component 2** | | | | | | | | | | |  |  |  |  |  |  |
| --- | --- | --- | --- | --- | --- | --- | --- | --- | --- | --- | --- | --- | --- | --- | --- | --- | --- | --- | --- | --- | --- | --- | --- | --- |
|  | **1^st^ tertile** | | **2^nd^ tertile** | | **3^rd^ tertile** | | **p for trend** | | **1^st^ tertile** | | **2^nd^ tertile** | | **3^rd^ tertile** | | | **p for trend** | |  |  |  |  |  |  |  |
|  | **(N=239)** | | **(N=238)** | | **(N=246)** | |  |  | **(N=239)** | | **(N=238)** | | **(N=246)** | | |  |  |  |  |  |  |  |  |  |
| **Sex** |  |  |  |  | |  | | | |  | |  | |  |  | |  | | | |  |  |  |  |
| Female | 45 (18.8%) | | 39 (16.4%) | | 49 (19.9%) | | 0.751 | | 61 (25.5%) | | 43 (18.1%) | | 29 (11.8%) | | | **<0.001** | | | |  |  |  |  |  |
| Male | 194 (81.2%) | | 199 (83.6%) | | 197 (80.1%) | |  | | 178 (74.5%) | | 195 (81.9%) | | 217 (88.2%) | | |  | | | |  |  |  |  |  |
| **Anthropometric measures** | | |  | |  | |  | |  | |  | |  | | |  | | | |  |  |  |  |  |
| Age, years | 56.0 [49.0;63.0] | | 59.0 [52.0;66.0] | | 59.0 [53.0;67.0] | | **<0.001** | | 58.0 [51.0;65.0] | | 58.0 [52.0;66.0] | | 58.0 [51.0;66.0] | | | 0.597 | | | |  |  |  |  |  |
| Body Mass Index, kg·m^-2^ | 26.0 [24.0;28.4] | | 27.9 [25.3;30.7] | | 29.7 [27.1;32.1] | | **<0.001** | | 26.9 [24.3;30.1] | | 28.4 [25.8;31.2] | | 28.2 [25.6;30.8] | | | **0.011** | | | |  |  |  |  |  |
| Waist-hip ratio | 0.97 [0.93;1.01] | | 0.98 [0.94;1.02] | | 1.00 [0.96;1.03] | | **<0.001** | | 0.98 [0.94;1.03] | | 0.98 [0.94;1.02] | | 0.98 [0.95;1.03] | | | 0.694 | | | |  |  |  |  |  |
| Neck circumference, cm | 39.0 [37.0;41.0] | | 41.0 [39.0;43.0] | | 41.0 [39.0;43.0] | | **<0.001** | | 40.0 [38.0;42.0] | | 40.0 [38.0;43.0] | | 41.0 [39.0;43.0] | | | **0.001** | | | |  |  |  |  |  |
| **Lifestyle risk factors** |  | |  | |  | |  | |  | |  | |  | | |  | | | |  |  |  |  |  |
| Smoking |  | |  | |  | | 0.367 | |  | |  | |  | | | 0.544 | | | |  |  |  |  |  |
| Never | 62 (25.9%) | | 63 (26.5%) | | 64 (26.0%) | |  | | 65 (27.2%) | | 66 (27.7%) | | 58 (23.6%) | | |  | | | |  |  |  |  |  |
| Former | 47 (19.7%) | | 54 (22.7%) | | 65 (26.4%) | |  | | 50 (20.9%) | | 58 (24.4%) | | 58 (23.6%) | | |  | | | |  |  |  |  |  |
| Current | 130 (54.4%) | | 121 (50.8%) | | 117 (47.6%) | |  | | 124 (51.9%) | | 114 (47.9%) | | 130 (52.8%) | | |  | | | |  |  |  |  |  |
| Drinking |  | |  | |  | | 0.344 | |  | |  | |  | | | 0.399 | | | |  |  |  |  |  |
| No | 187 (78.2%) | | 164 (68.9%) | | 183 (74.4%) | |  | | 182 (76.2%) | | 173 (72.7%) | | 179 (72.8%) | | |  | | | |  |  |  |  |  |
| Yes | 52 (21.8%) | | 74 (31.1%) | | 63 (25.6%) | |  | | 57 (23.8%) | | 65 (27.3%) | | 67 (27.2%) | | |  | | | |  |  |  |  |  |
| **Medical history** |  | |  | |  | |  | |  | |  | |  | | |  | | | |  |  |  |  |  |
| Hypertension | 84 (35.1%) | | 106 (44.5%) | | 135 (54.9%) | | **<0.001** | | 97 (40.6%) | | 112 (47.1%) | | 116 (47.2%) | | | 0.148 | | | |  |  |  |  |  |
| Diabetes mellitus | 35 (14.6%) | | 46 (19.3%) | | 60 (24.4%) | | **0.007** | | 50 (20.9%) | | 53 (22.3%) | | 38 (15.4%) | | | 0.126 | | | |  |  |  |  |  |
| Dyslipidemia | 189 (79.1%) | | 201 (84.5%) | | 206 (83.7%) | | 0.181 | | 183 (76.6%) | | 205 (86.1%) | | 208 (84.6%) | | | **0.022** | | | |  |  |  |  |  |
| Cerebrovascular disease | 3 (1.26%) | | 8 (3.36%) | | 5 (2.03%) | | 0.569 | | 7 (2.93%) | | 4 (1.68%) | | 5 (2.03%) | | | 0.506 | | | |  |  |  |  |  |
| Chronic pneumopathy | 7 (2.93%) | | 18 (7.56%) | | 10 (4.07%) | | 0.574 | | 13 (5.44%) | | 14 (5.88%) | | 8 (3.25%) | | | 0.259 | | | |  |  |  |  |  |
| Neurological disease | 5 (2.09%) | | 11 (4.62%) | | 15 (6.10%) | | **0.030** | | 13 (5.44%) | | 11 (4.62%) | | 7 (2.85%) | | | 0.158 | | | |  |  |  |  |  |
| Severity of ACS |  | |  | |  | | **0.012** | |  | |  | |  | | | 0.722 | | | |  |  |  |  |  |
| Mild | 223 (93.3%) | | 217 (91.2%) | | 213 (86.6%) | |  | | 217 (90.8%) | | 215 (90.3%) | | 221 (89.8%) | | |  | | | |  |  |  |  |  |
| Severe | 16 (6.69%) | | 21 (8.82%) | | 33 (13.4%) | |  | | 22 (9.21%) | | 23 (9.66%) | | 25 (10.2%) | | |  | | | |  |  |  |  |  |
| **Medication** |  | |  | |  | |  | |  | |  | |  | | |  | | | |  |  |  |  |  |
| Antihypertensive drug | 72 (30.1%) | | 99 (41.6%) | | 122 (49.6%) | | **<0.001** | | 86 (36.0%) | | 107 (45.0%) | | 100 (40.7%) | | | 0.302 | | | |  |  |  |  |  |
| Lipid-lowering drug | 54 (22.6%) | | 66 (27.7%) | | 68 (27.6%) | | 0.208 | | 62 (25.9%) | | 69 (29.0%) | | 57 (23.2%) | | | 0.480 | | | |  |  |  |  |  |
| Antidiabetic oral medication | 29 (12.1%) | | 33 (13.9%) | | 51 (20.7%) | | **0.009** | | 41 (17.2%) | | 42 (17.6%) | | 30 (12.2%) | | | 0.131 | | | |  |  |  |  |  |
| Insulin | 7 (2.93%) | | 8 (3.36%) | | 11 (4.47%) | | 0.361 | | 12 (5.02%) | | 8 (3.36%) | | 6 (2.44%) | | | 0.128 | | | |  |  |  |  |  |
| Antiplatelet and antithrombotic drugs | 20 (8.37%) | | 21 (8.82%) | | 28 (11.4%) | | 0.257 | | 29 (12.1%) | | 18 (7.56%) | | 22 (8.94%) | | | 0.236 | | | |  |  |  |  |  |
| **Cardiovascular variables** | | |  | |  | |  | |  | |  | |  | | |  | | | |  |  |  |  |  |
| Heart rate, bpm | 70.0 [63.5;80.0] | | 70.0 [62.0;78.0] | | 72.0 [64.0;80.0] | | 0.305 | | 70.0 [65.0;80.0] | | 71.0 [62.2;80.0] | | 70.0 [62.0;78.0] | | | 0.078 | | | |  |  |  |  |  |
| Systolic blood pressure, mmHg | 120 [111;132] | | 120 [110;136] | | 120 [110;132] | | 0.774 | | 119 [109;130] | | 121 [113;136] | | 121 [110;132] | | | 0.200 | | | |  |  |  |  |  |
| Creatinine, mg/dL | 0.82 [0.72;0.96] | | 0.86 [0.75;0.99] | | 0.84 [0.72;1.00] | | 0.317 | | 0.82 [0.70;0.99] | | 0.84 [0.75;0.96] | | 0.86 [0.77;1.03] | | | **0.004** | | | |  |  |  |  |  |
| Stents implanted | 214 (89.5%) | | 215 (90.3%) | | 223 (90.7%) | | 0.682 | | 213 (89.1%) | | 214 (89.9%) | | 225 (91.5%) | | | 0.386 | | | |  |  |  |  |  |
| Peak troponin, quartiles |  | |  | |  | | 0.243 | |  | |  | |  | | | 0.455 | | | |  |  |  |  |  |
| Quartile 1 | 41 (17.2%) | | 49 (20.6%) | | 40 (16.3%) | |  | | 41 (17.2%) | | 51 (21.4%) | | 38 (15.4%) | | |  | | | |  |  |  |  |  |
| Quartile 2 | 68 (28.5%) | | 58 (24.4%) | | 65 (26.4%) | |  | | 55 (23.0%) | | 67 (28.2%) | | 69 (28.0%) | | |  | | | |  |  |  |  |  |
| Quartile 3 | 70 (29.3%) | | 72 (30.3%) | | 61 (24.8%) | |  | | 64 (26.8%) | | 67 (28.2%) | | 72 (29.3%) | | |  | | | |  |  |  |  |  |
| Quartile 4 | 60 (25.1%) | | 59 (24.8%) | | 80 (32.5%) | |  | | 79 (33.1%) | | 53 (22.3%) | | 67 (27.2%) | | |  | | | |  |  |  |  |  |
| Cardiovascular event type |  | |  | |  | | 0.238 | |  | |  | |  | | | 0.931 | | | |  |  |  |  |  |
| Non-Q-wave AMI | 106 (44.4%) | | 97 (40.8%) | | 96 (39.0%) | |  | | 96 (40.2%) | | 102 (42.9%) | | 101 (41.1%) | | |  | | | |  |  |  |  |  |
| Unstable angina | 12 (5.02%) | | 12 (5.04%) | | 13 (5.28%) | |  | | 11 (4.60%) | | 17 (7.14%) | | 9 (3.66%) | | |  | | | |  |  |  |  |  |
| Q-wave AMI | 121 (50.6%) | | 129 (54.2%) | | 137 (55.7%) | |  | | 132 (55.2%) | | 119 (50.0%) | | 136 (55.3%) | | |  | | | |  |  |  |  |  |
| No recurrent CVE | 214 (89.5%) | | 211 (88.7%) | | 215 (87.4%) | |  | | 217 (90.8%) | | 212 (89.1%) | | 211 (85.8%) | | |  | | | |  |  |  |  |  |
| Recurrent CVE | 25 (10.5%) | | 27 (11.3%) | | 31 (12.6%) | |  | | 22 (9.21%) | | 26 (10.9%) | | 35 (14.2%) | | |  | | | |  |  |  |  |  |

The data are presented as the n (%) or median [25^th^ percentile; 75^th^ percentile]. Significant p values (p<0.05) are presented in bold. bpm: beats per minute; ACS: acute coronary syndrome; AMI: acute myocardial infarction; CVE: Cardiovascular event. Significant p values (p<0.05) are presented in bold.

##

## Table E8: Baseline characteristics by tertiles of respiratory polygraphy components in the HypnoLaus cohort.

|  | **Component 1** | | | | **Component 2** | | | |
| --- | --- | --- | --- | --- | --- | --- | --- | --- |
|  | **1^st^ tertile** | **2^nd^ tertile** | **3^rd^ tertile** | **p for trend** | **1^st^ tertile** | **2^nd^ tertile** | **3^rd^ tertile** | **p for trend** |
|  | **(N=640)** | **(N=640)** | **(N=660)** |  | **(N=640)** | **(N=640)** | **(N=)** |  |
| **Sex** |  |  |  |  |  |  |  |  |
| Female | 445 (69.5%) | 332 (51.9%) | 250 (37.9%) | **<0.001** | 326 (50.9%) | 286 (43.3%) | 1028 (53.0%) | **<0.001** |
| Male | 195 (30.5%) | 308 (48.1%) | 410 (62.1%) |  | 224 (35.0%) | 314 (49.1%) | 374 (56.7%) |  |
| **Anthropometric measures** | |  |  |  |  |  |  |  |
| Age, years | 50.8 [45.9;58.5] | 56.4 [49.4;67.1] | 65.5 [55.9;71.7] | **<0.001** | 55.7 [47.9;68.2] | 55.0 [48.3;66.9] | 61.2 [51.4;69.0] | **<0.001** |
| Body Mass Index, kg·m^-2^ | 23.4 [21.3;25.7] | 25.5 [23.4;28.0] | 27.8 [25.5;30.9] | **<0.001** | 25.4 [22.5;29.0] | 25.9 [23.5;28.6] | 25.4 [23.1;27.9] | 0.870 |
| Waist-hip ratio | 0.9 [0.8;0.9] | 0.9 [0.9;1.0] | 0.9 [0.9;1.0] | **<0.001** | 0.9 [0.9;1.0] | 0.9 [0.9;1.0] | 0.9 [0.9;1.0] | **0.006** |
| Neck circumference, cm | 34.0 [32.0;37.0] | 36.5 [34.0;39.0] | 39.0 [36.0;41.0] | **<0.001** | 35.5 [33.0;39.0] | 37.0 [34.0;40.0] | 37.0 [34.0;39.5] | **<0.001** |
| **Lifestyle risk factors** |  |  |  |  |  |  |  |  |
| Smoking |  |  |  | **0.009** |  |  |  | **0.001** |
| Never | 281 (43.9%) | 284 (44.4%) | 241 (36.5%) |  | 255 (39.8%) | 271 (42.3%) | 281 (42.6%) |  |
| Former | 229 (35.8%) | 243 (38.0%) | 291 (44.1%) |  | 233 (36.4%) | 253 (39.5%) | 277 (42.0%) |  |
| Current | 124 (19.4%) | 107 (16.7%) | 118 (17.9%) |  | 146 (22.8%) | 110 (17.2%) | 92 (13.9%) |  |
| Weekly alcohol consumption, units | 3.0 [1.0;7.0] | 4.0 [1.0;9.0] | 5.0 [2.0;11.0] | **<0.001** | 3.0 [1.0;8.0] | 4.0 [1.0;9.0] | 4.5 [2.0;10.0] | **0.01** |
| **Medical history** |  |  |  |  |  |  |  |  |
| Hypertension | 143 (22.3%) | 231 (36.1%) | 385 (58.3%) | **<0.001** | 225 (35.2%) | 255 (39.8%) | 279 (42.3%) | **0.032** |
| Diabetes mellitus | 17 (2.7%) | 48 (7.5%) | 108 (16.4%) | **<0.001** | 54 (8.4%) | 59 (9.2%) | 60 (9.1%) | 0.866 |
| Dyslipidemia | 99 (15.5%) | 177 (27.7%) | 233 (35.3%) | **<0.001** | 170 (26.6%) | 192 (30.0%) | 146 (22.1%) | **0.005** |
| **Medication** |  |  |  |  |  |  |  |  |
| Antihypertensive drug | 79 (12.3%) | 127 (19.8%) | 257 (38.9%) | **<0.001** | 144 (22.5%) | 153 (23.9%) | 166 (25.2%) | 0.533 |
| Lipid lowering drug | 70 (10.9%) | 108 (16.9%) | 188 (28.5%) | **<0.001** | 122 (19.1%) | 125 (19.5%) | 118 (17.9%) | 0.734 |
| Antidiabetics oral medication | 8 (1.2%) | 26 (4.1%) | 55 (8.3%) | **<0.001** | 30 (4.7%) | 30 (4.7%) | 29 (4.4%) | 0.958 |
| Insulin | 1 (0.2%) | 6 (0.9%) | 12 (1.8%) | **0.01** | 7 (1.1%) | 4 (0.6%) | 8 (1.2%) | 0.526 |
| **Cardiovascular variables** | |  |  |  |  |  |  |  |
| New cardiovascular event* | 12 (1.9%) | 20 (3.1%) | 43 (6.5%) | **<0.001** | 24 (3.8%) | 28 (4.4%) | 23 (3.5%) | 0.695 |
| Heart rate**, bpm | 61.6 [56.9;66.6] | 62.2 [56.5;67.0] | 63.2 [57.8;68.7] | **<0.001** | 63.4 [58.1;68.3] | 62.4 [57.5;67.6] | 61.0 [56.2;65.9] | **<0.001** |
| Systolic blood pressure, mmHg | 117.0 [107.5;128.5] | 124.5 [113.5;136.5] | 130.5 [120.9;142.0] | **<0.001** | 122.0 [111.0;135.5] | 124.0 [114.0;136.0] | 126.5 [115.0;138.0] | **<0.001** |

The data are presented as the n (%) or median [25^th^ percentile; 75^th^ percentile]. Significant p values (p<0.05) are presented in bold. bpm: beats per minute. Significant p values (p<0.05) are presented in bold.

* CV events: any non-fatal CV event (stroke, heart attack, CABG or PCI) from PSG to follow up 2

**Average heart rate during sleep

## Table E9. Cox proportional hazard for the primary composite endpoint in the ISAACC cohort considering both components and the individual respiratory polygraphy parameters.

| **ALL (N=723)** | **HR* (95% CI)** | **p value** |
| --- | --- | --- |
| **Components from PCA**** |  |  |
| Component 1 | 0.82 (0.6-1.13) | 0.222 |
| Component 2 | 1.38 (0.99-1.94) | 0.0598 |
| **Respiratory polygraphy parameters** | |  |
| AHI, events per h | 1.01 (0.99-1.02) | 0.235 |
| ODI > 4%, per h | 1 (0.99-1.01) | 0.982 |
| Mean SaO_2_, % | 1.08 (0.97-1.2) | 0.175 |
| Minimum SaO_2_, % | 1.05 (1.01-1.09) | 0.0157 |
| Time with SaO_2_ < 90%, % | 0.99 (0.98-1.01) | 0.288 |
| Average duration of events, sec | 1.01 (0.97-1.04) | 0.745 |

*Adjusted for age, sex, smoking, alcohol consumption, obesity, hypertension, stroke, diabetes, ACS severity, dyslipidemia, antihypertensive drugs, antiplatelet and antithrombotic drugs, heart rate, systolic blood pressure, creatinine, stents implanted, cardiovascular event type and troponin peak. **Additionally, adjusted for mean SaO_2_

## Table E10: Cox proportional hazards model for the primary composite endpoint in the ISAACC cohort.

|  | **All**  **(N=723)** | |
| --- | --- | --- |
|  | **HR* (95% CI)** | **p value** |
| **Crude** |  |  |
| AHI < 15 events/h | 1 |  |
| AHI ≥15 events/h | **1.7 (1.07–2.7)** | **0.026** |
| **Adjusted*** |  |  |
| AHI < 15 events/h | 1 |  |
| AHI ≥15 events/h | 1.62 (0.98–2.67) | 0.0577 |

HR (95% CI): hazard ratio (95% confidence interval). All variables included in the model met the assumptions for proportional hazards. Significant p values (p<0.05) are presented in bold. *Adjusted for age, sex, smoking, alcohol consumption, obesity, hypertension, stroke, diabetes, ACS severity, dyslipidemia, antihypertensive drugs, antiplatelet and antithrombotic drugs, heart rate, systolic blood pressure, creatinine, stents implanted, cardiovascular event type and troponin peak and mean SaO_2_. ACS: Acute coronary syndrome; AHI: apnea-hypopnea Index; CVD: Cardiovascular disease.

# References

1. Chiner E, Arriero JM, Signes-Costa J, et al. Validación de la versión española del test de somnolencia Epworth en pacientes con síndrome de apnea de sueño [Validation of the Spanish version of the Epworth Sleepiness Scale in patients with a sleep apnea syndrome]. Arch Bronconeumol 1999; 35: 422–427.
